# Supplementary figures and images for: lra: A long read aligner for sequences and contigs
Source: PLoS Comput Biol. 2021 Jun 21;17(6):e1009078. doi: 10.1371/journal.pcbi.1009078 (PMC8248648; doi:10.1371/journal.pcbi.1009078)

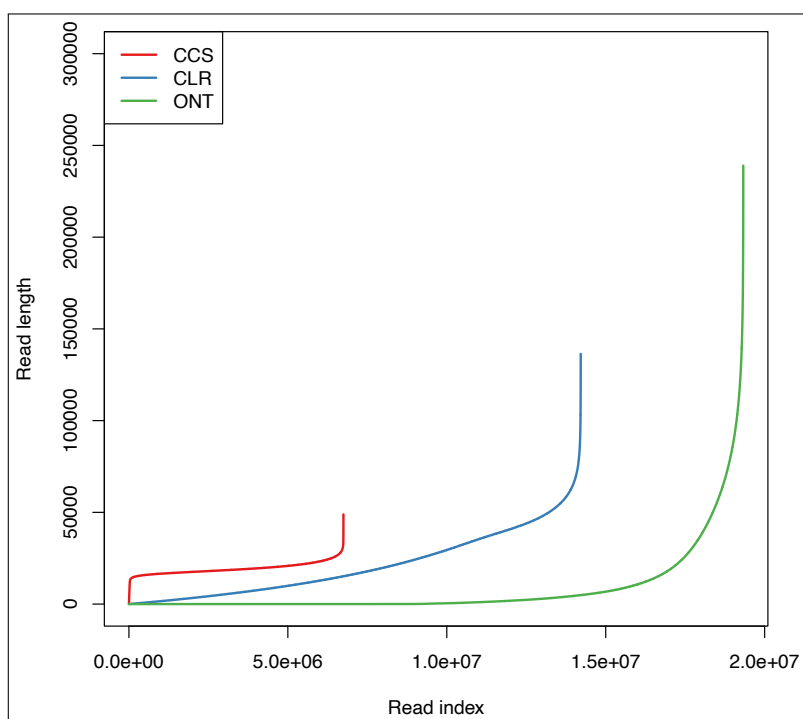

Figure S1: The distribution of read lengths from the HG002 HiFi, CLR, and ONT data.

Supplement: S1 Fig — (PDF) [file pcbi.1009078.s001.pdf]

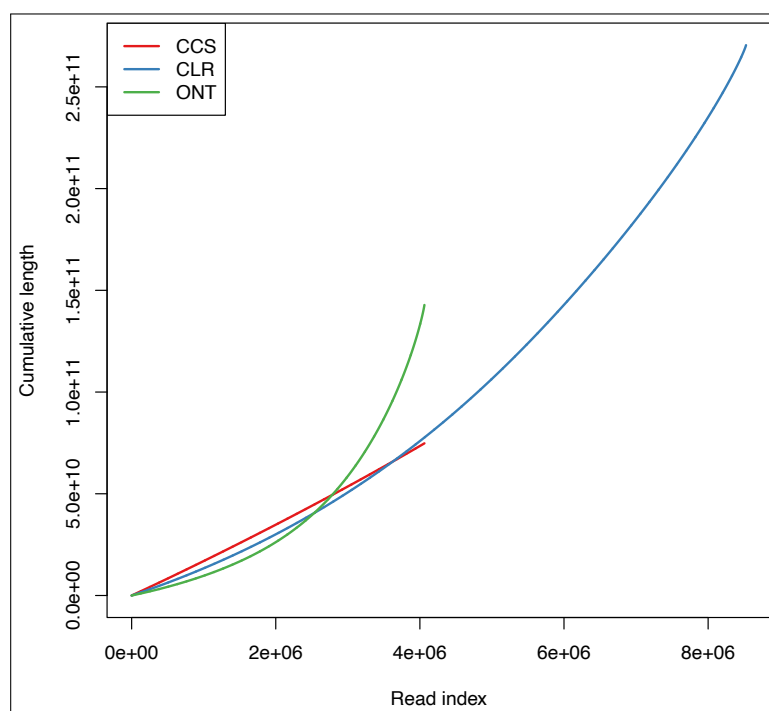

Figure S2: The cumulative number of bases from the HiFi, CLR, and ONT data.

Supplement: S2 Fig — (PDF) [file pcbi.1009078.s002.pdf]
