# Supplementary material for: lra: A long read aligner for sequences and contigs
Source: PLoS Comput Biol. 2021 Jun 21;17(6):e1009078. doi: 10.1371/journal.pcbi.1009078 (PMC8248648; doi:10.1371/journal.pcbi.1009078)
Supplement: S3 Fig — The data structures for each subproblem: DI, DV, DP, EI, EV, EP, EL, EB and the process of subproblems solving. The horizontal axis represents the query, while the vertical axis represents the target. Points are numbered in Cartesian sorted order, which is the processing order. 12 points are assigned into three column subproblems (A0c,B0c), (A1c,B1c), (A2c,B2c) and one row subproblem (A0r,B0r), where starting points are assigned to A-part and endpoints are assigned to B-part. Leaf subproblems are not shown for simplicity. Start and End are used for the trace-back of the optimal chain. Start stores sub—the index of the subproblem which yields the optimal chaining score up to a starting point and ind—the index of fi in array EI, that is φ(EI, fi), where fi is the diagonal of the starting point. End stores the optimal value for each endpoint. For this toy example, gap cost of appending fragment αj to fragment αi is gap(αi,αj)=0.25*log(|(yie-xie)-(yjs-xjs)+1|)+1, where (xie,yie) is the endpoint of αi and (xjs,yjs) is the startpoint of αj. m, shows the regions that each subproblem covers and the initialized data structures for each subproblem. There are of three column subproblems and one row subproblems (leaf subproblems are not shown for simplicity): (A0c,B0c), (A1c,B1c), (A2c,B2c) and (A0r,B0r). a-l shows how the data structures of subproblems that are associated with the point being processed in each step are updated. Note that entries that are updated are highlighted by orange. a, shows for startpoint − 1, it is a leaf subproblem that yields the value of the optimal chain up to startpoint − 1. b, shows when processing endpoint − 2, the optimal value up to it is Score(startpoint − 1) + 2, where 2 is the match bonus of the fragment. Array End is then updated. Since endpoint − 2 is in A0c, A1c, A0r, the corresponding DV entries are updated to 2 and corresponding DP entries update to the index of endpoint − 2. c, shows when processing startpoint − 3, it is in the B-part [file pcbi.1009078.s003.pdf]

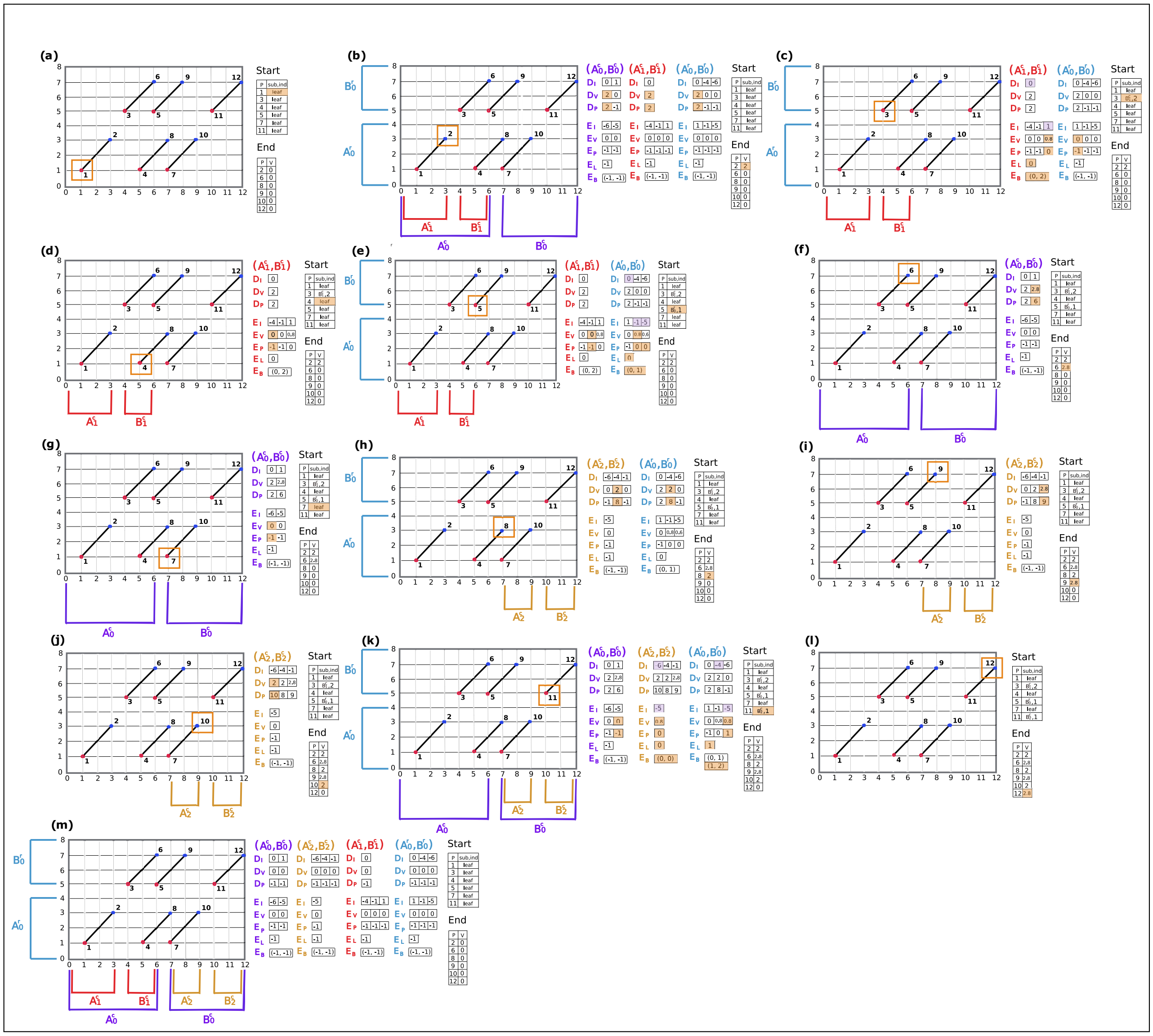

(a)
(b)
(c)
(d)
(e)
(f)
(g)
(h)
(i)
(j)
(k)
(l)
(m)
Start
End
P
sub.ind
leaf
V

Figure S3: A detailed example of visualizing subproblems division, the data structures for each subproblem: D_I, D_V, D_P, E_I, E_V, E_P, E_L, E_B and the process of subproblems solving. The horizontal axis represents the query, while the vertical axis represents the target. Points are numbered in Cartesian sorted order, which is the processing order. 12 points are assigned into three column subproblems (A_0^c, B_0^c), (A_1^c, B_1^c), (A_2^c, B_2^c) and one row subproblem (A_0^r, B_0^r), where starting points are assigned to A-part and endpoints are assigned to B-part. Leaf subproblems are not shown for simplicity. Start and End are used for the trace-back of the optimal chain. *Start* stores *sub* – the index of the subproblem which yields the optimal chaining score up to a starting point and *ind* – the index of f_i in array E_I, that is $\varphi(E_I, f_i)$, where f_i is the diagonal of the starting point. *End* stores the optimal value for each endpoint. For this toy example, gap cost of appending fragment α_j to fragment α_i is $gap(\alpha_i, \alpha_j) = 0.25 * log\left(\left|(y_i^e - x_i^e) - (y_j^s - x_j^s) + 1\right|\right) + 1$, where (x_i^e, y_i^e) is the endpoint of α_i and (x_j^s, y_j^s) is the startpoint of α_j. **m**, shows the regions that each subproblem covers and the initialized data structures for each subproblem. There are of three column subproblems and one row subproblems (leaf subproblems are not shown for simplicity): (A_0^c, B_0^c), (A_1^c, B_1^c), (A_2^c, B_2^c) and (A_0^r, B_0^r). **a-l** shows how the data structures of subproblems that are associated with the point being processed in each step are updated. Note that entries that are updated are highlighted by orange. **a**, shows for $startpoint - 1$, it is a leaf subproblem that yields the value of the optimal chain up to $startpoint - 1$. **b**, shows when processing $endpoint - 2$, the optimal value up to it is $Score(startpoint - 1) + 2$, where 2 is the match bonus of the fragment. Array *End* is then updated. Since $endpoint - 2$ is in A_0^c, A_1^c, A_0^r, the corresponding D_V entries are updated to 2 and corresponding D_P entries update to the index of $endpoint - 2$. **c**, shows when processing $startpoint - 3$, it is in the B-parts of subproblems (A_1^c, B_1^c) and (A_0^r, B_0^r). $startpoint - 3$ is located in $E_I[2]$ of (A_1^c, B_1^c), so $Update(D_V[0], E_B)$ would be called to get the value of $E_V[2]$. The purple color highlighting shows what forward diagonals in E_V would be updated by $D_V[0]$. $E_P[2]$ would be updated to point to $D_I[0]$. $startpoint - 3$ is located in $E_I[2]$ of (A_0^r, B_0^r) and no forward diagonals in D_I used to update $E_V[2]$. Therefore, in *Start*, *sub* and *ind* for $startpoint - 3$ are updated to $B_1^c, 2$. **d**, shows when processing $startpoint - 4$, it is in B_1^c and locates in $E_I[0]$ of (A_1^c, B_1^c). Since there is no forward diagonal in D_I can be used to update $E_V[0]$, it is a leaf subproblem that yields the optimal chaining value up to $startpoint - 4$ in *Start*. **e**, shows when processing $startpoint - 5$, it is in B_1^c and B_0^r. In (A_1^c, B_1^c), there is no forward diagonal can be used to update $E_V[1]$. In (A_0^r, B_0^r), $Update(D_V[0], E_B)$ is called to update the block structure E_B, so $E_V[1]$ and $E_V[2]$ would be computed from E_B. In *Start*, *sub* and *ind* for $startpoint - 5$ are updated to $B_0^r, 1$. **f**, **g**, **h**, **i**, **j**, **k**, **l**: show the subproblems solving and data structures updating for the rest of points. After processing all 12 points, three optimal chains can be obtained by tracing back, which are $chain - 1 = [startpoint - 1, endpoint - 2, startpoint - 3, endpoint - 6]$, $chain - 2 = [startpoint - 1, endpoint - 2, startpoint - 5, endpoint - 9]$ and $chain - 3 = [startpoint - 7, endpoint - 10, startpoint - 11, endpoint - 12]$.
