# Supplementary material for: lra: A long read aligner for sequences and contigs
Source: PLoS Comput Biol. 2021 Jun 21;17(6):e1009078. doi: 10.1371/journal.pcbi.1009078 (PMC8248648; doi:10.1371/journal.pcbi.1009078)
Supplement: S1 Text — (PDF) [file pcbi.1009078.s004.pdf]

We analyzed the use of a local minimizer index for alignments of 263,000 PacBio CLR reads from HG002 chromosome 20 and simulated HiFi, CLR, and ONT reads.

Although banded alignment is used to refine alignments in each case, the alignments that skip the additional chaining step with local minimizer matches have an 11.5-fold increase in medium size (10-50 base) deletions and 3.8-fold increase in medium sized insertions compared to alignments that use the local minimizer index (Table A). Although this effect could be minimized using a larger band when refining alignments, this would have a much higher computational burden.

Table A: The count of indels from PacBio CLR alignments.

Using local index	Matches	Deletions (all)	Deletions 10-50 bp	Insertions (all)	Insertions 10-50 bp
Yes	5.58B	198M	114K	170M	972K
No	5.47B	216M	1.31M	200M	3.67M

To further investigate the impact that local minimizers have on mapping quality, we measured the accuracy of alignments of simulated reads with and without the local minimizer index (Fig A).

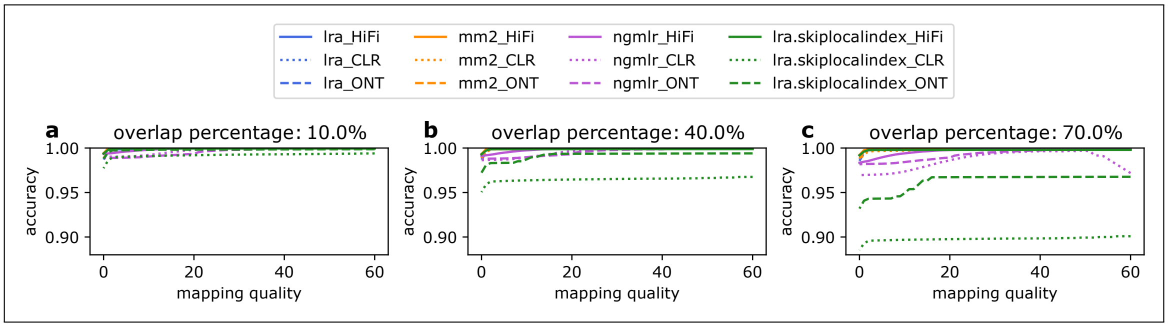

Figure A: Mapping accuracy of lra, lra without the step of refining local minimizers, minimap2 and ngmlr on simulated HiFi, CLR reads for lengths between 5-50kb and ONT reads for lengths between 1-50kb. Simulated reads were mapped to genome GRCh38. A read is considered as correctly mapping if the reported mapping interval has $\geq 10\%, 40\%, 70\%$ overlap with the truth interval. *paftools.js mapeval* was used to evaluate the mapping accuracy.
