# Supplementary material for: lra: A long read aligner for sequences and contigs
Source: PLoS Comput Biol. 2021 Jun 21;17(6):e1009078. doi: 10.1371/journal.pcbi.1009078 (PMC8248648; doi:10.1371/journal.pcbi.1009078)
Supplement: S2 Text — (PDF) [file pcbi.1009078.s005.pdf]

Compared to read alignment, assembly alignment has the simplicity of detecting variants directly from assembly alignments rather than forming a consensus of SV signatures from multiple reads. To gauge the specificity of assembly-based calls with no raw reads alignments support, we used *Truvari bench* to find large SV calls (>= 20000 bases) only in each assembly haplotype alignment but not in HiFi/CLR read pbsv-calls for lra and minimap2 respectively. We display the counts of assembly exclusive large SV calls for both lra and minimap2 (Table B) and the genome coordinates of all such calls (Table C and A). We also show several IGV screenshot where the assembly alignments show clear insertions/deletions in regions with no read alignment pbsv-calls (Fig A and B)).

Table A: minimap2 assembly exclusive large SV calls coordinates. We used *Truvari bench* to find large SV calls (>= 20000 bases) only in each haplotype alignment but not in HiFi/CLR read pbsv-calls for minimap2. All the genome coordinates of such calls are given below.

minimap2	
hap1	hap2
11:3674982-3705654	8:145091981-145111902
X:154976-173756	-
8:145091981-145111902	-

Table B: Assembly exclusive large SV calls counts. We used *Truvari bench* to find large SV calls (>= 20000 bases) only in each assembly haplotype alignment but not in HiFi/CLR read pbsv-calls for lra and minimap2 respectively.

	lra	minimap2
hap1	53	3
hap2	60	1

Table C: lra assembly exclusive large SV calls counts. We used *Truvari bench* to find large SV calls (>= 20000 bases) only in each haplotype alignment but not in HiFi/CLR read pbsv-calls for lra. All the genome coordinates of such calls are given below.

lra	
hap1	hap2
3:57387036-57435371	12:8558485-8590846
20:54123233-54164549	7:98387266-98418964
11:3292907-3338726	7:142486372-142506795
11:3675340-3706579	21:11097542-11160785
11:18963939-18986081	21:14369638-14407903
11:70801579-70840687	18:65121221-65141721
8:2250148-2266001	15:75549963-75574240
8:6868084-6887194	X:3794818-3833246
8:86780666-86808014	X:114959695-114989535
8:86795475-86819747	X:148906424-148953566
8:145091981-145111902	X:153482307-153520111
15:102292812-102314488	2:19195289-19213771
7:74962806-74990794	2:92269995-92294337
7:98387266-98418978	2:169727087-169744070
7:142486372-142506798	9:73334694-73352687
13:57714736-57734449	9:137041193-137067504
19:7515550-7532254	3:57387036-57435372
19:34883088-34900034	3:195232056-195249800
19:37793776-37839249	4:9254709-9278459
19:55345568-55361211	4:49282710-49300465
14:106094430-106114188	4:132678548-132698553
4:49122506-49143435	8:145091981-145111902
22:23852646-23876941	5:21481673-21502808
5:12685942-12706067	5:70068238-70088683
5:21481923-21503055	5:94550118-94566747
5:69511859-69527482	10:27605760-27653051
5:70067784-70108676	10:37467951-37491453
5:94550118-94566748	13:112957399-112975655
16:33293751-33330123	1:2606562-2622757
2:19206009-19224510	1:110224909-110243353
2:92269995-92294314	1:121363555-121385437
2:169727087-169744065	1:143236902-143265608
9:73316730-73334723	1:148012547-148037641
9:135950709-135971104	1:148277867-148319102
Y:6559132-6574907	1:148290499-148319089
Y:22280073-22329633	1:207702938-207721493
X:231384-270899	14:106089337-106108921
X:2649604-2699486	14:106800271-106824194
6:80084902-80112738	19:7019185-7037577
6:161033867-161067185	19:7061694-7080819
12:8574169-8594807	19:7515550-7533066
12:10575957-10591368	19:21755939-21803995
1:16892289-16912661	19:34883088-34900034
1:121397737-121416393	19:36793371-36836323
1:146421568-146451608	19:55345858-55362099
1:207702938-207721493	11:3268153-3313942
1:246982645-247000283	11:18963939-18986084
10:27605760-27653054	11:60973285-60992117
10:45837663-45853704	11:70801579-70840683
21:11097544-11113501	11:87688378-87734401
21:14369638-14407887	20:54123233-54164712
21:15258940-15288279	22:23852646-23876916
18:65121221-65141610	6:58142577-58165006
-	6:80084902-80112738
-	6:157732494-157765464
-	6:161064125-161108458
-	16:32297406-32325807
	16:32525817-32551589
-	16:33237891-33254312
-	16:33248007-33293676

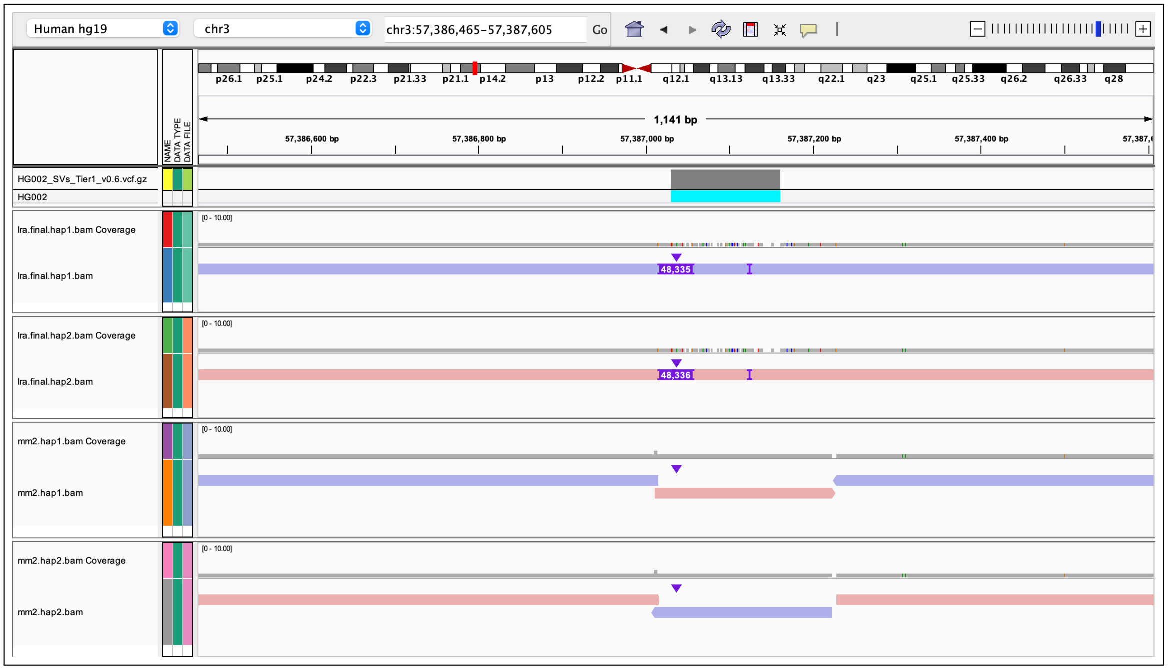

Figure A: The top track shows the GIAB HG002 curated SV set. The next 2 tracks are lra alignment of HG002 hifiasm assembly two haplotypes and bottom two tracks are minimap2 alignment. There is a clear insertion of 48336 bases in lra alignment, which also appears in the GIAB HG002 curated SV set. minimap2 alignment shows a inversion instead at the same location. This large insertion is missing in the HiFi and CLR read alignment.

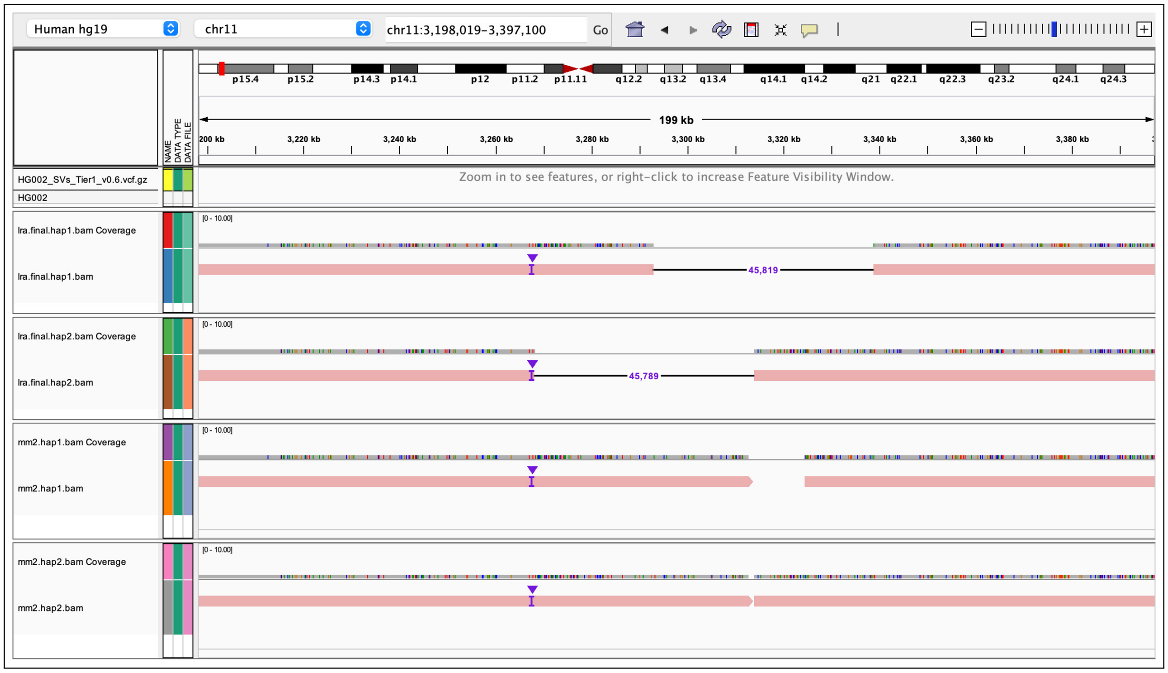

Figure B: The top track shows the GIAB HG002 curated SV set. The next 2 tracks are lra alignment of HG002 hifiasm assembly two haplotypes and bottom two tracks are minimap2 alignment. There is a clear insertion of 48789 bases in lra alignment, which also appears in the GIAB HG002 curated SV set. minimap2 alignment splits the contig at the same location. This large deletion is missing in the HiFi and CLR read alignment.
