# Supplementary material for: lra: A long read aligner for sequences and contigs
Source: PLoS Comput Biol. 2021 Jun 21;17(6):e1009078. doi: 10.1371/journal.pcbi.1009078 (PMC8248648; doi:10.1371/journal.pcbi.1009078)
Supplement: S1 Algorithm — (PDF) [file pcbi.1009078.s006.pdf]

Algorithm S1 Defining subproblems

Require: X - the sorted set of starting and ending points from the set of anchors Φ; $[s, e]$ - the column/row range; $d \in \{col, row\}$;

Ensure: SUB(d) - the desired set of column/row subproblems obtained by assigning points from column/row s to column/row e, which is initialized as $\emptyset$;

procedure SUB(d, s, e, X)
  if $e == s$ **then**
    Construct subproblem $(d, \emptyset, \emptyset, s, e,$DATA) with DATA= $(\emptyset, \emptyset, \emptyset, E_I, E_P, E_V)$ as following:
    Save the forward diagonals of all the starting points from column/row e in array E_I;
    Sort the forward diagonals in array E_I in the increasing/decreasing order;
    Initialize every entry in E_V to 0;
    Initialize every entry in E_P to -1;
    SUB(d) ←SUB(d) $\cup \{(d, \emptyset, \emptyset, s, e,$DATA$)\}$;
  else
    while $e > s$ **do**
      Construct subproblem $(d, s, \lfloor (s+e)/2 \rfloor, \lfloor (s+e)/2 \rfloor + 1, e,$DATA) with DATA= $(D_I, D_P, D_V, E_I, E_P, E_V)$ as following:
      Store the forward diagonals of all the ending points from column/row s to column/row $\lfloor (s+e)/2 \rfloor$ in array D_I;
      Sort the forward diagonals in array D_I in the increasing/decreasing order;
      Initialize every entry in D_V to 0;
      Initialize every entry in D_P to -1;
      Save the forward diagonals of all the starting points from column/row $\lfloor (s+e)/2 \rfloor + 1$ to column/row e in array E_I;
      Sort the forward diagonals in array E_I in the increasing order;
      Initialize every entry in E_V to 0;
      Initialize every entry in E_P to -1;
      SUB(d) ←SUB(d) $\cup \{(d, s, \lfloor (s+e)/2 \rfloor, \lfloor (s+e)/2 \rfloor + 1, e,$DATA$)\}$;
      SUB($d, s, \lfloor (s+e)/2 \rfloor, X$);
      SUB($d, \lfloor (s+e)/2 \rfloor + 1, e, X$);
  return SUB(d);
