# Supplementary material for: lra: A long read aligner for sequences and contigs
Source: PLoS Comput Biol. 2021 Jun 21;17(6):e1009078. doi: 10.1371/journal.pcbi.1009078 (PMC8248648; doi:10.1371/journal.pcbi.1009078)
Supplement: S2 Algorithm — (PDF) [file pcbi.1009078.s007.pdf]

Algorithm S2 Sparse Dynamic Programming with convex gap cost

```
Get the points set X from the set of anchors Φ;
Sort X in Cartesian order; Assume all points are arranged from col 0 to col t;
SUB← ∅;                                   ▷ SUB is the set storing all subproblems
SUB←SUB∪Sub(col, 0, t, X);
Sort X in anti-Cartesian order; Assume all points are arranged from row 0 to row q;
SUB←SUB∪Sub(row, 0, q, X);
for each p_i ∈ CartesianSort(X) do
    if p_i is an endpoint then
        Score(p_i) ← Score(p_s) + l_i, where p_s is the corresponding startpoint and l_i is match
bonus of the corresponding anchor;
        End(p_i) ← Score(p_i);        ▷ End stores the optimal chaining score for each endpoint
        for each SUB[j]∈ SA do
            if Score(p_i) > D_V[j] then
                D_P[j] ← i;
                D_V[j] ← Score(p_i), where j = φ(D_I, f_i) and f_i is the forward diagonal of p_i;
    else if p_i is a starting point then
        maxvalue ← 0;
        for each SUB[h] ∈ SB do
            j ← φ(E_I, f_i), where f_i is the forward diagonal of p_i;
            if SUB[h] is a col-based subproblem then
                if D_I[E_L] > f_i then
                    E_V[j] ← Ω(E_B, j);
                else
                    for each k ∈ (E_L, C), where C = min_s D_I[s] > f_i do
                        Update(D_V[k], E_B);
                    E_L ← C − 1;
                    E_V[j] ← Ω(E_B, j);
            else if SUB[h] is a row-based subproblem then
                if D_I[E_L] ≤ f_i then
                    E_V[j] ← Ω(E_B, j);
                else
                    for each k ∈ (E_L, C), where C = min_s D_I[s] ≤ f_i do
                        Update(D_V[k], E_B);
                    E_L ← C − 1;
                    E_V[j] ← Ω(E_B, j);
            if E_V[j] > maxvalue then
                Start[p_i] ← (SUB[h], j)
```
