# Supplementary material for: lra: A long read aligner for sequences and contigs
Source: PLoS Comput Biol. 2021 Jun 21;17(6):e1009078. doi: 10.1371/journal.pcbi.1009078 (PMC8248648; doi:10.1371/journal.pcbi.1009078)
Supplement: S1 Table — Considering HiFi/CLR + pbsv callsets and ONT + Sniffles callsets, the size of the intersection of every two callsets are shown. The intersection is calculated using Truvari bench on two called vcfs. (PDF) [file pcbi.1009078.s008.pdf]

Table S1: Shared variant calls. Considering HiFi/CLR + pbsv callsets and ONT + Sniffles callsets, the size of the intersection of every two callsets are shown. The intersection is calculated using *Truvari bench* on two called vcfs.

	HiFi			CLR			ONT		
	lra	pbmm2	ngmlr	lra	pbmm2	ngmlr	lra	pbmm2	ngmlr
lra	22159	20469	17776	22319	19868	18162	23170	15375	14092
pbmm2	20469	21430	17826	19869	21748	18344	15371	21338	14104
ngmlr	17777	17827	18779	18165	18344	19981	14091	14107	18598
