# Supplementary material for: lra: A long read aligner for sequences and contigs
Source: PLoS Comput Biol. 2021 Jun 21;17(6):e1009078. doi: 10.1371/journal.pcbi.1009078 (PMC8248648; doi:10.1371/journal.pcbi.1009078)
Supplement: S2 Table — (PDF) [file pcbi.1009078.s009.pdf]

Table S2: Calls unique to a callset in tandem repeats and segmental duplications. Entry with pbmm2 as row and lra as column means the number of unique calls in pbmm2 callset when comparing pbmm2 callset and lra callset.

	HiFi			CLR			ONT		
	lra	pbmm2	ngmlr	lra	pbmm2	ngmlr	lra	pbmm2	ngmlr
lra	0	948	988	0	1811	1778	0	5899	4477
pbmm2	1677	0	938	2384	0	1610	7743	0	4469
ngmlr	4368	3588	0	4113	3376	0	9060	7203	0
In tandem repeats									
lra	0	344	414	0	799	809	0	2510	2315
pbmm2	483	0	391	899	0	748	2934	0	2228
ngmlr	1251	1090	0	1479	1322	0	3177	2683	0
In segmental duplications									
lra	0	385	155	0	509	302	0	1083	367
pbmm2	944	0	150	912	0	318	1960	0	403
ngmlr	1398	833	0	1291	903	0	2440	1600	0
