# Supplementary material for: lra: A long read aligner for sequences and contigs
Source: PLoS Comput Biol. 2021 Jun 21;17(6):e1009078. doi: 10.1371/journal.pcbi.1009078 (PMC8248648; doi:10.1371/journal.pcbi.1009078)
Supplement: S3 Table — SVs: Indels (insertions and deletions), inversions were simulated by SUVIVOR. All SVs were simulated by SUVIVOR. HiFi and CLR reads were simulated by PBSIM and ONT reads were simulated by alchemy2. We simulated 195 Indels (insertions and deletions) of lengths between 50-10000 bases, 97 inversions of lengths between 600-2000 bases. (PDF) [file pcbi.1009078.s010.pdf]

Table S3: Comparison of the Truvari result between all combinations of aligners and SV callers on simulated HiFi, CLR and ONT dataset with simulated SVs: indels (insertions and deletions), inversions. All SVs were simulated by SUVIVOR. HiFi and CLR reads were simulated by PBSIM and ONT reads were simulated by alchemy2, which is distributed with lra source. We simulated 195 Indels (insertions and deletions) of lengths between 50-10000 bases, 97 inversions of lengths between 600-2000 bases.

	HiFi																	
	INDEL									INV								
	pbsv			cuteSv			sniffles		.	pbsv			cuteSv			sniffles		
aligner	lra	pbmm2	ngmlr	lra	minimap2	ngmlr	lra	minimap2	ngmlr	lra	pbmm2	ngmlr	lra	minimap2	ngmlr	lra	minimap2	ngmlr
TP base	**195**	191	191	**195**	194	193	171	**188**	185	**90**	**90**	89	**97**	96	96	**97**	95	96
TP call	**195**	191	191	**195**	194	193	171	**188**	185	**90**	**90**	89	**195**	182	183	**97**	95	96
FP	**0**	4	**0**	**0**	2	6	6	**1**	**1**	**0**	**0**	2	**1**	5	3	3	**2**	4
FN	**0**	4	4	**0**	1	2	24	**7**	10	**7**	**7**	8	**0**	1	1	**0**	2	1
precision	**1**	0.979	**1**	**1**	0.990	0.970	0.966	**0.995**	**0.995**	**1**	**1**	0.978	**0.995**	0.973	0.984	0.970	**0.979**	0.960
recall	**1**	0.979	0.979	**1**	0.995	0.990	0.877	**0.964**	0.949	**0.928**	**0.928**	0.917	**1**	0.990	0.990	**1**	0.980	0.990
F1 score	**1**	0.979.	0.990	**1**	0.992	0.980	0.919	**0.980**	0.971	**0.963**	**0.963**	0.946	**0.997**	0.981	0.987	**0.985**	0.979	0.975

	CLR																	
	INDEL									INV								
	pbsv			cuteSV			sniffles			pbsv			cuteSV			sniffles		
aligner	lra	pbmm2	ngmlr	lra	minimap2	ngmlr	lra	minimap2	ngmlr	lra	pbmm2	ngmlr	lra	minimap2	ngmlr	lra	minimap2	ngmlr
TP base	**195**	190	193	**195**	**195**	193	154	179	**181**	**90**	**90**	87	96	**97**	95	96	**97**	94
TP call	**195**	190	193	**195**	**195**	193	155	179	**181**	**90**	**90**	87	**195**	**195**	189	**99**	97	94
FP	**0**	**0**	3	**0**	3	2	**3**	8	5	**0**	**0**	3	2	**0**	3	74	**2**	**2**
FN	**0**	5	2	**0**	**0**	2	41	**9**	14	**7**	**7**	10	1	**0**	2	2	**0**	3
precision	**1**	**1**	0.985	**1**	0.985	0.990	**0.981**	0.957	0.973	**1**	**1**	0.967	0.990	**1**	0.994	0.572	**0.980**	0.979
recall	**1**	0.974	0.990	**1**	**1**	0.990	0.790	0.918	**0.928**	**0.928**	**0.928**	0.897	0.990	**1**	0.948	0.990	**1**	0.969
F1 score	**1**	0.987	0.987	**1**	0.992	0.990	0.875	0.937	**0.950**	**0.963**	**0.963**	0.930	0.990	**1**	0.971	0.725	**0.990**	0.974

	ONT											
	INDEL						INV					
	cuteSV			sniffles			cuteSV			sniffles		
	lra	minimap2	ngmlr	lra	minimap2	ngmlr	lra	minimap2	ngmlr	lra	minimap2	ngmlr
TP base	188	**191**	189	140	143	**155**	**97**	**97**	96	80	93	**95**
TP call	188	**191**	189	140	143	**155**	**193**	**193**	192	83	93	**95**
FP	3	**2**	4	**3**	6	17	**2**	**2**	3	35	**1**	10
FN	7	**4**	6	55	52	**40**	**0**	**0**	1	17	4	**2**
precision	0.984	**0.990**	0.979	**0.979**	0.959	0.901	**0.990**	**0.990**	0.985	0.703	**0.989**	0.9047
recall	0.964	**0.979**	0.969	0.718	0.733	**0.795**	**1**	**1**	0.990	0.825	0.959	**0.979**
F1 score	0.974	**0.985**	0.974	0.828	0.831	**0.845**	**0.995**	**0.995**	0.987	0.759	**0.974**	0.940
