# Supplementary material for: lra: A long read aligner for sequences and contigs
Source: PLoS Comput Biol. 2021 Jun 21;17(6):e1009078. doi: 10.1371/journal.pcbi.1009078 (PMC8248648; doi:10.1371/journal.pcbi.1009078)
Supplement: S4 Table — Complicated nested SVs: deletion-inversion-deletions (INVDEL), inverted-duplications (INVDUP), which were simulated by SUVIVOR. HiFi and CLR reads were simulated by PBSIM and ONT reads were simulated by alchemy2, which is distributed with lra source. We simulated 100 deletion-inversion-deletions and inverted-duplications of lengths between 600-1000 bases respectively. Inversion-deletion is a type of nested SV where an inversion is flanked by 2 deletions and inversion-duplication means the duplicated sequence is inverted. For deletion-inversion-deletions, the inversion and two deletions have all been found in order to be counted as a TP. For cases where the inversion and only one flanked deletion are found were counted as Partial. For inverted-duplications, both the inversion and duplication need to be found in order to be counted as a TP. We found that minimap2 alignment find inverted-duplications as insertions, therefore, we didn’t count that as TP. (PDF) [file pcbi.1009078.s011.pdf]

Table S4: Comparison of the Truvari result between all combinations of aligners and SV callers on simulated HiFi, CLR and ONT dataset with complicated nested SVs: deletion-inversion-deletions (INVDEL), inverted-duplications (INVDUP), which were simulated by SUVIVOR. HiFi and CLR reads were simulated by PBSIM and ONT reads were simulated by alchemy2, which is distributed with lra source. We simulated 100 deletion-inversion-deletions and inverted-duplications of lengths between 600-1000 bases respectively. Inversion-deletion is a type of nested SV where an inversion is flanked by 2 deletions and inversion-duplication means the duplicated sequence is inverted. For deletion-inversion-deletions, the inversion and two deletions have all been found in order to be counted as a TP. For cases where the inversion and only one flanked deletion are found were counted as Partial. For inverted-duplications, both the inversion and duplication need to be found in order to be counted as a TP. We found that minimap2 alignment find inverted-duplications as insertions, therefore, we didn't count that as TP.

| | INVDEL | | | | | | | | | INVDUP | | | | | | | | |
|---|---|---|---|---|---|---|---|---|---|---|---|---|---|---|---|---|---|---|
| | HiFi | | | CLR | | | ONT | | | HiFi | | | CLR | | | ONT | | |
| aligner | lra | minimap2 | ngmlr | lra | minimap2 | ngmlr | lra | minimap2 | ngmlr | lra | minimap2 | ngmlr | lra | minimap2 | ngmlr | lra | minimap2 | ngmlr |
| TP | **98** | 93 | 93 | 1 | **94** | 74 | **37** | 1 | 2 | 94 | 0 | **98** | **99** | 0 | 97 | **100** | 58 | **98** |
| Partial | 2 | 5 | 6 | 79 | 6 | 21 | 62 | 97 | 97 | - | - | - | - | - | - | - | - | - |
| FN | **0** | 2 | 1 | 20 | **0** | 5 | **1** | 2 | **1** | 6 | 100 | **2** | **1** | 100 | 3 | **0** | 42 | 2 |
