# Supplementary material for: lra: A long read aligner for sequences and contigs
Source: PLoS Comput Biol. 2021 Jun 21;17(6):e1009078. doi: 10.1371/journal.pcbi.1009078 (PMC8248648; doi:10.1371/journal.pcbi.1009078)
Supplement: S5 Table — The breakpoints accuracy analysis was conducted by comparing the boundaries of true positive SVs from Truvari result to the curated SVs’ breakpoints for each aligner/caller combination. Breakpoints accuracy is measured by the percentage of SVs with perfect breakpoint boundaries and the average shifting distance between the left-most coordinate of SV boundaries. (PDF) [file pcbi.1009078.s012.pdf]

Table S5: Comparison of the breakpoints on real datasets. The breakpoints accuracy analysis was conducted by comparing the boundaries of true positive SVs from Truvari result to the curated SVs' breakpoints for each aligner/caller combination. Breakpoints accuracy is measured by the percentage of SVs with perfect breakpoint boundaries and the average shifting distance between the left-most coordinate of SV boundaries.

	pbsv						sniffles								
	HiFi			CLR			HiFi			CLR			ONT		
	lra	minimap2	ngmlr	lra	minimap2	ngmlr	lra	minimap2	ngmlr	lra	minimap2	ngmlr	lra	minimap2	ngmlr
SVs% with zero breakpoint shifting	64	64	65	59	58	60	38	68	6	33	55	7	31	57	6
Average breakpoint shifting distance(bp)	45.6	42.44	38.07	47.38	47.00	35.00	52.82	38.42	56.98	59.68	40.49	40.38	60.93	41.25	52.94
Total TP	9466	9408	8433	9438	9414	9076	8924	8700	8015	8969	8082	6533	9085	8887	8471
