# Supplementary material for: lra: A long read aligner for sequences and contigs
Source: PLoS Comput Biol. 2021 Jun 21;17(6):e1009078. doi: 10.1371/journal.pcbi.1009078 (PMC8248648; doi:10.1371/journal.pcbi.1009078)
Supplement: S6 Table — The breakpoints accuracy analysis was conducted by comparing the boundaries of true positive SVs to the bondaries of ground truth SV for each aligner/caller combination. Breakpoints accuracy is measured by the percentage of SVs with perfect breakpoint boundaries and the average shifting distance between the left-most coordinate of SV boundaries. (PDF) [file pcbi.1009078.s013.pdf]

Table S6: Comparison of the breakpoints on simulated SVs: indels, inversions, deletion-inversion-deletions, inverted-duplications. The breakpoints accuracy analysis was conducted by comparing the boundaries of true positive SVs to the bondaries of ground truth SV for each aligner/caller combination. Breakpoints accuracy is measured by the percentage of SVs with perfect breakpoint boundaries and the average shifting distance between the left-most coordinate of SV boundaries.

	INDEL								
	HiFi			CLR			ONT		
aligner	lra	minimap2	ngmlr	lra	minimap2	ngmlr	lra	minimap2	ngmlr
total TP	171	**188**	185	154	179	**181**	140	143	**155**
SV% of zero shifting distance of breakpoint	**84.2**	81.9	51.9	**72.1**	**72.1**	42.0	**75.0**	74.8	48.4
average shifing distance of breakpoint	**0.25**	**0.25**	0.78	0.37	**0.37**	1.10	1.72	**0.90**	0.93

	INV								
	HiFi			CLR			ONT		
aligner	lra	minimap2	ngmlr	lra	minimap2	ngmlr	lra	minimap2	ngmlr
total TP	**97**	95	96	95	**97**	94	**95**	93	**95**
SV% of zero shifting distance of breakpoint	5.2	**6.3**	**6.3**	8.4	**33.0**	26.6	**27.4**	20.4	**27.4**
average shifing distance of breakpoint	4.40	1.19	**1.15**	7.97	**0.80**	0.84	3.02	0.97	**0.92**

	INVDEL								
	HiFi			CLR			ONT		
aligner	lra	minimap2	ngmlr	lra	minimap2	ngmlr	lra	minimap2	ngmlr
total TP	**298**	287	289	161	**294**	264	**235**	197	200
SV% of zero shifting distance of breakpoint	7.8	15.4	**19.9**	1.1	**20.3**	19.4	11.3	**15.8**	9.1
average shifing distance of breakpoint	279.11	**261.16**	284.40	302.18	278.75	**238.12**	272.72	**238.48**	285.09

	INVDUP								
	HiFi			CLR			ONT		
aligner	lra	minimap2	ngmlr	lra	minimap2	ngmlr	lra	minimap2	ngmlr
total TP	189	100	**198**	176	100	**195**	**200**	155	198
SV% of zero shifting distance of breakpoint	12.7	**78.0**	27.8	33.5	**79.0**	30.3	19.0	**57.4**	26.3
average shifing distance of breakpoint	4.38	**0.27**	1.37	3.04	**0.32**	0.82	1.61	**0.48**	0.90
