# Supplementary material for: lra: A long read aligner for sequences and contigs
Source: PLoS Comput Biol. 2021 Jun 21;17(6):e1009078. doi: 10.1371/journal.pcbi.1009078 (PMC8248648; doi:10.1371/journal.pcbi.1009078)
Supplement: S7 Table — Truvari comparisons between lra, minimap2 and ngmlr using the Genome in a Bottle benchmark SV set. Optimal results in each category are shown in bold. TP-base means true positive calls in the benchmark SV curation set, while TP-call means true positive calls in the SV set from each aligner. False positive means the number of non-matching calls from the SV set from each aligner. False negative means the number of non-matching calls from the SV curation set. (PDF) [file pcbi.1009078.s014.pdf]

Table S7: Truvari classification of cuteSV variant calls. Truvari comparisons between lra, minimap2 and ngmlr using the Genome in a Bottle benchmark SV set. Optimal results in each category are shown in bold. TP-base means true positive calls in the benchmark SV curation set, while TP-call means true positive calls in the SV set from each aligner. False positive means the number of non-matching calls from the SV set from each aligner. False negative means the number of non-matching calls from the SV curation set.

	cuteSV								
	HiFi			CLR			ONT		
	lra	minimap2	ngmlr	lra	minimap2	ngmlr	lra	minimap2	ngmlr
TP base	**9402**	9370	9190	4103	4139	**8980**	9303	**9314**	9256
TP call	**9386**	9360	9182	4100	4158	**8980**	9293	**9344**	9249
FP	**456**	562	1448	243	217	1101	**774**	1003	1282
FN	**239**	271	451	5538	5502	**661**	338	**327**	385
precision	**0.954**	0.943	0.863	0.944	**0.95**	0.891	**0.923**	0.903	0.878
recall	**0.975**	0.972	0.953	0.426	0.4293	**0.931**	0.965	**0.966**	0.96
F1 score	**0.964**	0.957	0.906	0.587	0.5914	**0.91**	**0.944**	0.934	0.917
