# Supplementary material for: lra: A long read aligner for sequences and contigs
Source: PLoS Comput Biol. 2021 Jun 21;17(6):e1009078. doi: 10.1371/journal.pcbi.1009078 (PMC8248648; doi:10.1371/journal.pcbi.1009078)
Supplement: Attachment — Submitted filename: LRA Review comments.pdf [file pcbi.1009078.s015.pdf]

We have made the following changes to the analysis of alignment and SV calling, which have changed many statistics in the manuscript. All differences are minor, and the primary results, that the use of concave-gap sparse dynamic programming effectively discovers structural variation on long reads and assembled contigs.

1. We report differences between the number of calls only across pbsv callsets, to simplify accounting.
2. We use Truvari 'bench' to calculate overlap between datasets. This decreases the number of calls considered overlapping 1-15%, depending on the data that are compared, but we feel the comparison is more standardized in this manner. Default parameters are used for the benchmark operation.
3. We have simplified our comparison of where callsets differ, highlighting the genomic features of calls unique to a dataset.
4. We have changed from evaluating alignments of the more recent hifiasm genome, which has affected the numbers of SV called.
5. To simplify inversion curation, we rely only on comparison to the hifiasm.
6. We include another SV caller cuteSV except pbsv and sniffles, since we want to compare the SV calling results under different callers, considering the possible bias in callers.
7. We conduct simulation studies on insertions, deletions, inversions and nested SVs, like inverted-duplications and deletion-inversion-deletions.
8. We conduct breakpoint analysis on simulation datasets as well as real datasets.
9. We change the simulator for simulating Pacbio reads to PBSIM, which is more popularly used.

Reviewer #1: Ren & Chaisson described lra, a new long-read aligner specialized for calling structural variations (SVs). Lra implements a chaining algorithm with concave gap cost functions. Although the algorithm is not new, as the authors said, this is probably the first time the algorithm gets implemented. This manuscript represents an important addition in this field. Critical observations are as follows:

COMMENT 1.

1) I tried lra v1.1.0 on reads simulated by PBSIM. At mapping quality 60, lra wrongly mapped 1.3% of reads. This is not as good as NGMLR. While simulation based evaluation may not reflect the accuracy on real data, it is a good sanity check and indicative of low-level algorithmic issues. I wonder if the authors can improve the mapping accuracy further. The file is available at https://github.com/lh3/minimap2/releases/download/v2.0/pb-1.fa.gz and the mapping result can be evaluated by "paftools.js mapeval -r 1e-6".

RESPONSE 1.

Thank you for this suggestion. After modifying our algorithm to account for translocations and other complex rearrangements (COMMENT 19), we used the evaluation of mapping accuracy on simulated reads to discover low-level algorithmic issues, which improved alignment statistics overall. We used pbsim to generate 158603/322673 reads of CCS, CLR data, and used alchemy2 (included with lra source) to generate 1683696 ONT reads, and mapped to GRCh38 (excluding alternate haplotypes). Because our current estimate of mapping quality has a wider spread, we test mapping accuracy on reads with mapqv at least 20 , and find the false mapping rates to be 0.11%, 0.03%, 0.03% for the respective data types, when a 40% overlap with the simulated interval is required for a correct alignment .

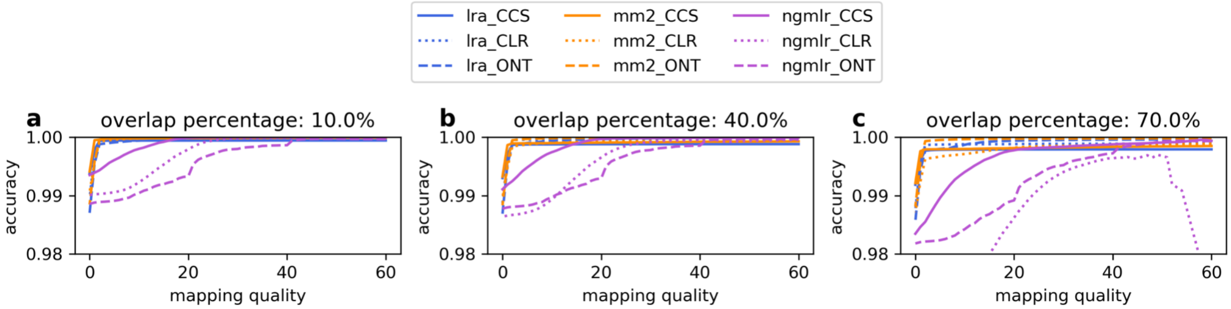

COMMENT 2.

2) For a second test, I mapped the HG002 maternal hifiasm assembly to GRCh38 and call variants with "htsbox -vcf ref.fa -q5 -S10000 -T20 srt.bam | awk '$6==1'". Minimap2 -xasm5 calls 3.75M variants, which is about right. Lra calls 9.53M. The number is too high, suggesting the majority are false positives. I guess this is caused by inaccurate base alignment. I understand the authors are focusing on SVs, but the high false positive rate of small variants is concerning. It would be good if the authors can try to improve that.

RESPONSE 2.

The number of variants were driven by a combination of the small insertions and deletions allowed by the sparse dynamic programming and a non-affine dynamic programming alignment used to fill some gaps. We have added an alignment step that uses affine-gap alignment to refine contig alignments once the rough alignment is generated.

This refining gives the following variant counts for the hifiasm contigs:

aligner		NA24385.HiFi.hifiasm-0.12.hap1.fa	NA24385.HiFi.hifiasm-0.12.hap2.fa
	total variants	3.77M	3.86M
lra			

	# of variant >= 50b	15638	16128
	# of 1b < variant < 50b	0.84M	0.85M
	total variants	3.64M	3.74M
	# of variant >= 50b	16400	16886
mm2	# of 1b < variant < 50b	0.67M	0.699M

The differences between the two methods may now be as a result of alignment scoring rather than problems in our approach.

COMMENT 3.

3) For each query sequence, minimap2 chains all anchors in one round and produces multiple chains. In comparison, lra clusters anchors first and then chains in each cluster. While lra chaining may be theoretically better, clustering is heuristic and may not be as good. Combining clustering and chaining might hurt overall mapping accuracy. The authors said they use clustering "for efficiency". How much efficiency is affected if we use lra chaining for all anchors?

RESPONSE 3.

We agree that an optimal approach to mapping sequences would not involve clustering anchors prior to a chaining operation. However, because the CG-SDP chaining has a relatively large constant factor for each step of calculation, the application of a clustering step that removes anchors that are extremely unlikely to take part in an alignment does result in significant time improvements. Alignment of CLR reads from human chromosome 20 requires 11 minutes using 40 cores when pre-clustering anchors and discarding anchors not in clusters, and roughly ⅕ of the data was aligned without clustering in a similar time. When clustering prior to chaining, roughly 2.9% of anchors are retained for chaining. By measuring the alignment accuracy and SV calling, we show that the clustering approach does not remove informative anchors at a level that affects alignment or SV calling.

The primary cause for alignment errors in comment 1 was that missed portions of alignments at the beginning and ending of reads caused reads to be mapped to incorrect repeats of the genome, and the primary cause for insertions and deletions identified in comment 2 is inherent to the method used to refine alignments after chaining, neither of which are affected by the clustering routine.

Minor comments

COMMENT 4.

4) Minimap2+paftools is primarily developed for calling small variants, not for calling SVs. We can increase the recall of SV calling by adding options "-r2k -z2000,200" or even "-r7k -z2000,200". This trades performance and small variant accuracy for SV accuracy.

RESPONSE 4.

Previously we used paftools.js to call SVs for Minimap2 assembly alignment. We now change to htsbox pileup to call SVs from Ira and Minimap2 HG002 hifiasm alignment. We also used Minimap2+paftools.js with the parameter options as suggested :“-r2k -z2000,200” and "-r7k -z2000,200". The f1 score using htsbox pileup is greater than paftools.js with either of the specified parameters. We used truvari to compare the assembly alignment calls with GIAB curated SV sets and the result is presented in the table below. Ira has slightly higher precision, recall and f1 score.

	Ira+htsbox	mm2+htsbox	mm2+paftools 2k	mm2+paftools 7k
TP	9310	9035	8962	8962
FP	546	692	666	666
FN	331	606	679	679
precision	0.9451	0.92905	0.931	0.931
recall	0.9656	0.9371	0.9295	0.9295
F1 score	0.9553	0.93308	0.93029	0.93029
total call	25982	26341	25037	25037

Because of the decreased statistics for the suggested parameters of paftools.js, we elected to use htsbox to call variants.

COMMENT 5.

5) The manuscript is using concave gap cost functions, not convex.

RESPONSE 5.

We agree with this comment. Previously, after deliberation we had elected to use convex since this was used by Gusfield despite the confusion. We have changed the usage of convex to concave.

COMMENT 6.

6) Apparently, Ira requires C++14 or C++17 to compile. However, Makefile is not using an option like -std=c++14. I got a compilation error.

RESPONSE 6.

We have updated our default build command to specify c++14.

Reviewer: Heng Li

Reviewer #2: Long read sequencing is a technology that becomes more important in recent days. Alignment of long reads is the fundamental step for many downstream analyses. In this manuscript, the authors described the design of Ira, and compared the output to other programs. The tool is extreme valuable for long reads data analyses. It is challenging to understand all details described in the manuscript, but the paper is well-written, solid and with enough details. I also tested the program. It is easy to install and use. For an actively emerging area, any new tools are welcomed, and Ira is one of the excellent ones from my point of view. Thus, I suggest accepting the manuscript with some minor revisions as shown below. I suggest the authors to improve the GitHub page to make it easier for users to understand the benefit of using Ira.

Minor points:

COMMENT 7.

Page 2 line 47, change “translocation” to “translocations”.

RESPONSE 7.

This has been updated.

COMMENT 8.

Page 5 line 150, change “section 3.3” to something else, as currently there is no title like “3.3”. The same for other similar cases.

RESPONSE 8.

This has been changed to simply not reference a section.

COMMENT 9.

There are a lot of abbreviations in this paper, such as “DATA” (line 206), “SA”, “SB” (line 237). It is better to add some explanations to make it easier for readers to follow. SA means subproblem A part?

RESPONSE 9.

In section 3.6.1, we have descriptions of abbreviations in a list format, which we included hoping to make the method easier to follow. This list contains the components of the data structure referenced by DATA, and we now reference this list when describing the data structure.

The statement:

> Each subproblem is described by the label d ∈ {column, row}, the starting and ending rows and columns of the subproblem A.s, A.e, B.s, B.e, and a data structure of values used to compute the optimal chain, DATA.

has been updated as:

> Each subproblem is described by the label d ∈ {column,row}, the starting and ending rows and columns of the subproblem A.s, A.e, B.s, B.e, and a set of lists collectively referenced as DATA that are used in the calculation of optimal chains, as described in 3.6.1.

Furthermore, we changed the descriptions of SA and SB to be in list format.

COMMENT 10.

Line 343, change "can be only be" to "can only be".

RESPONSE 10.

This has been updated.

COMMENT 11.

Line 413, not a problem but I am surprised that GRCh37 instead of GRCh38 is used. Maybe because the curated variant file is based on GRCh37? Why It is "mapped to GRCh38" in line 427?

RESPONSE 11.

The curated variant file is based on GRCh37, however this build contains limited centromeric and satellite DNA. We align simulated data to GRCh38 in order to evaluate mapping accuracy with the added challenge of satellite DNA. We have included the additional statement in the results describing why simulated data is aligned to GRCh38:

> The simulated data were mapped to GRCh38 to evaluate mapping accuracy with a genome that includes centromeric sequences.

COMMENT 12.

Line 423, “the number of bases aligned by Ira and minimap2 are within 0.03-7%”. I do not understand. Do you mean the difference between the alignments by the two programs?

RESPONSE 12.

We refer to the difference. To clarify our statement, we have updated it to:

> The difference of bases aligned by Ira and minimap2 are within 0.03-7%.

Have all data underlying the figures and results presented in the manuscript been provided?

Large-scale datasets should be made available via a public repository as described in the *PLOS Computational Biology* data availability policy, and numerical data that underlies graphs or summary statistics should be provided in spreadsheet form as supporting information.

Reviewer #1: Yes

Reviewer #2: Yes

PLOS authors have the option to publish the peer review history of their article (what does this mean?). If published, this will include your full peer review and any attached files.

If you choose “no”, your identity will remain anonymous but your review may still be made public.

Do you want your identity to be public for this peer review? For information about this choice, including consent withdrawal, please see our Privacy Policy.

Reviewer #1: **Yes:** Heng Li

Reviewer #2: No

Reviewer 3.

Review of “Ira: A long read aligner for sequences and contigs”
Overview: Ren et. al. has presented an alignment algorithm for long reads and contigs, and its application in detection of structural variations in the manuscript “Ira: A long read aligner for sequence and contigs”. The algorithm performs sparse dynamic programming with convex gap functions on the clusters of anchors. The authors have shown that Ira has competitive runtime and memory usage to that of minimap2. Although the alignment accuracy with Ira (on simulated data) is slightly less than the alignemnt accuracy with minimap2, Ira can map sequences across long indel variants andtherefore, the results show an increase in the sensitivity of SV calls, especially, with Oxford Nanopore (ONT) data.

Verdict: The paper presents some interesting ideas, especially the use of convex fap functions in sparse dymanic programming that has benefits in SV calling. However, a significant revision is advocated taking into account the following comments.

The specific comments are as follows.

COMMENT 13.

1. The authors discuss about the additional local minimizer indexing which are parameterized by minimizer parameters k l and w l , and a tiling window W l . However, the significance of this step is not clear.

RESPONSE 13.

We have expanded our description of the motivation for using local minimizer indexes with the following text:

> The chains of anchors for low-accuracy single-pass SMS reads maybe sparse or have spurious off-diagonal matches in repetitive sequences.The detailed alignment may be calculated using dynamic programming within the region that is chained, however to limit the computation required for refining the alignment we implement an additional index of local minimizers that are used to refine an alignment once a coarse-chaining has been done. The local index is parameterized by $k_l < k$, $w_l < w$, and a tiling length W_l and is composed of a collection of separate minimizer indexes sequence intervals of length W_l tiling across both the query and the target.

The utility of the local minimizer index was measured by comparing the alignment results using the local index versus relying on global minimizer chaining alone. While we recognize the results of this test are influenced by the specific implementation of Ira, the following results motivate the use of the local index:

- Alignment accuracy on simulated CLR and ONT data drops 3-9% when alignments are not refined using the local index.
- There is an 11.5-fold increase of the number of medium-length deletions (10-50 bases) and 3.8-fold increase in the number of medium-length insertions when alignments are not refined using the local minimizer index.

We have added the following analysis to the supplement.

With the step of refining by local minimizers, the mapping accuracy of reads with mapqv at least 20 is 99.89%, 99.97% and 99.97% for CCS, CLR and ONT respectively, when a 40% overlap with the simulated interval is required for a correct alignment (More details in RESPONSE 17).

Without the step of refining by local minimizers, for simulated CCS data, the mapping accuracy has almost no difference on all 10%, 40%, 70% overlap percentage metrics. A great drop of mapping accuracy happened for simulated CLR and ONT data. When evaluating using 70% overlap percentage metric, the mapping accuracy for CLR and ONT even drops below 0.90 and 0.97 respectively, which indicates the step of refining by local minimizers is vital to correctly mapping reads with relatively higher error rate. Therefore, the step of refining by local minimizer is used for high error rate read types.

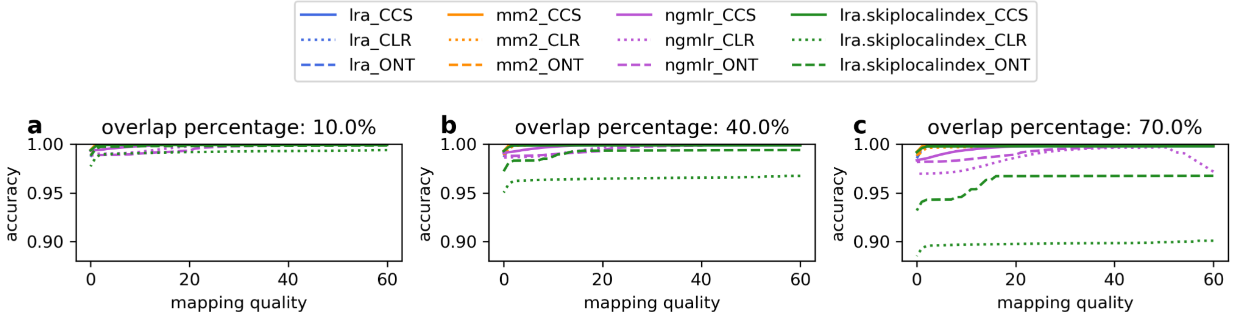

COMMENT 14.

2. In the clustering step of the algorithm, how are D R and D F parameters determined in the Algorithm?

RESPONSE 14.
The value of D^R is chosen so that the majority of spurious anchors are not used in CG-SDP by filtering during rough clustering, and was determined empirically. We have added the following statement to the methods to clarify this:

> The value of D^R is chosen so that rough clusters are likely to contain at least minClusterSize true anchors from a read (default to 3 for CLR/ONT, and 10 for

HiFi/contigs). For CLR and ONT data, we empirically determined a sufficient D^R is 200, and 150 for HiFi and assembly contig alignment.

D^F is used during fine clustering to merge close unique anchors together, and to fill in non-unique anchors between unique anchors to form fine clusters. The objective of fine clustering is to have clusters represent linear alignments without SV nor repetition. A large value of D^F would allow linear alignments over repetitive sequences (e.g. satellite DNA) to be merged into a single cluster. A value of D^F that is small would over fragment clusters. We empirically determined D^F=500 is sufficient to align CCS sequences and contigs.

We have added the following statement to clarify how D^F is chosen:

> In the step of fine clustering, we empirically found D^F=500 was able to distinguish clustering anchors in different tandem repeats and allowed a sufficient number of repetitive anchors to be included in the fine clusters.

COMMENT 15.

3. The alignment analysis is mainly done for simulated long reads (5 − 50 kb). But the average read length for ONT data might be around 1 − 2 kb. Therefore, it is not clear how the aligner would perform for the reads that are 1−5 kb long (that may be spanning a SV). In many typical ONT datasets, the read lengths are much longer ≈ 5 − 20kb, how do these methods perform on such datasets if available for SV?

RESPONSE 15.

We have updated the simulation for ONT reads with length between 1-50kb in the mapping accuracy and SV simulation analysis. The mapping accuracy result is shown in RESPONSE 17. The SV simulation result is shown in RESPONSE 19.

In our manuscript, we have performed analysis using three real sequencing datasets all from the Genome in a Bottle NA24385 genome: a PacBio CCS dataset, a PacBio CLR dataset, and an ONT dataset. These datasets have median lengths of, 18,794, 16,395, and 321 bases, respectively, which agrees with the comment regarding a bias towards shorter reads when considering a statistic by the number of reads. However, when considering the minimum read length such that 90% of the bases in the dataset are contained by that read length or greater (e.g. n90 read length), these values are 16,390, 11,845, and 7589 bases for the CCS, CLR, and ONT datasets. This indicates that long (>7kb) reads contribute the vast majority of the coverage for the ONT data, despite a large number of short reads. The distribution of the read lengths has been added to the supplementary material, replicated below.

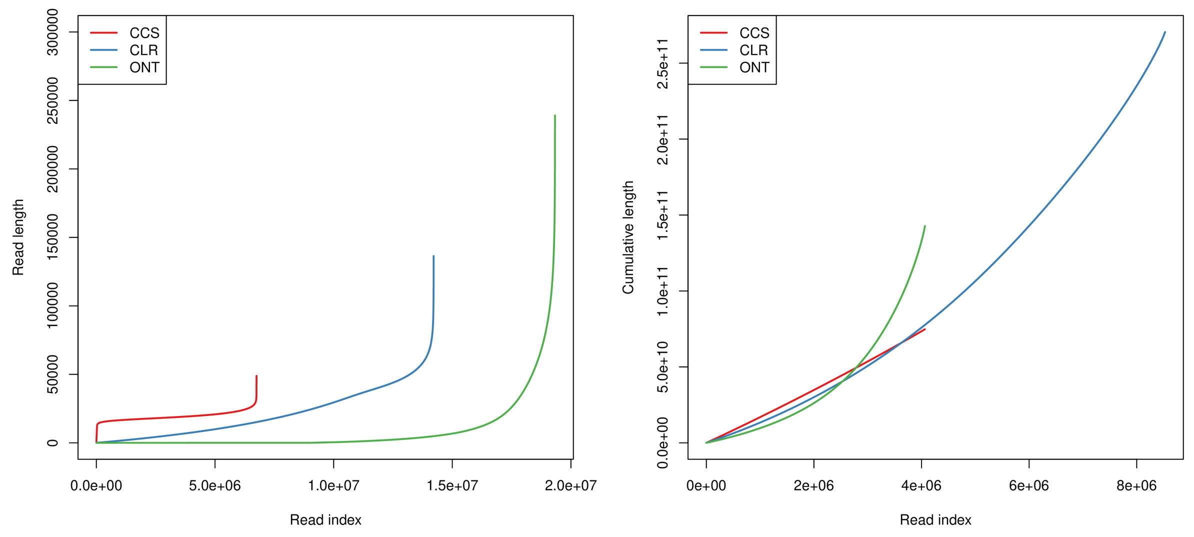

COMMENT 16.

4. It is unclear how the alignment accuracy is computed for the simulated data? What is the metric used? Please provide the details.

RESPONSE 16.

We measure alignment accuracy using simulated data, and comparing the coordinates of the true simulated intervals and the aligned intervals for each read. When a read is mapped to reference, it can be split into several segments due to noisy pieces in the read, with each segment mapped to a location. We use the metric "if the longest alignment of a read has more than a certain overlap with the true interval, we consider it as correctly mapping". This metric is widely used by other aligners like minimap2. We also measured the mapping accuracy under three overlap percentage thresholds: 10%, 40%, 70%, for the respective data types. We've added the description of mapping accuracy analysis to the manuscript:

> we compared the mapping accuracy of lra, minimap2 and ngmlr on simulated HiFi, CLR data for read lengths between 5-50kb and ONT data for read lengths between 1-50kb. HiFi and CLR reads were simulated by PBSIM (https://github.com/pfaucon/PBSIM-PacBio-Simulator) and ONT data were simulated by a model-based simulator, alchemy2, that is distributed with the lra source. Reads were mapped GRCh38 and considered as correctly mapping if the reported mapping interval has >= 10% overlap with the truth interval. paftools.js mapeval was used to evaluate the mapping accuracy. Three overlap percentages (10%, 40%, 70%) were used to measure the change of mapping accuracy with overlap percentage evaluation metric. The mapping accuracy of reads with mapqv at least 20 is 99.89%, 99.97% and 99.97% for

HiFi, CLR and ONT respectively, when a 40% overlap with the simulated interval is required for a correct alignment (Figure 1). As the overlap percentage goes up from 10% to 70%, the mapping accuracy of lra alignment decreases 0.1%, 0.09%, 0.03% for HiFi, CLR and ONT at mapqv 20 due to differences in lengths of sequences aligned in repetitive regions.

COMMENT 17.

5. The metric used for considering an alignment as a correct mapping is “the longest alignment has more than 30% overlap with the simulated interval”. However, what is the reasoning behind the choice of this threshold? How does the percentage of correct mapping change with the choice of this threshold for lra?

RESPONSE 17.
We have changed our mapping evaluation to use three different thresholds, 10%, 40%, and 70%. The results have been reproduced below.

The mapping accuracy of reads with mapqv at least 20 is 99.89%, 99.97% and 99.97% for CCS, CLR and ONT respectively, when a 40% overlap with the simulated interval is required for a correct alignment. As the overlap percentage goes up from 10% to 70%, the mapping accuracy of lra alignment decreases 0.1%, 0.09%, 0.03% for CCS, CLR and ONT at mapqv 20 due to possible reads alignment split across structural variants.

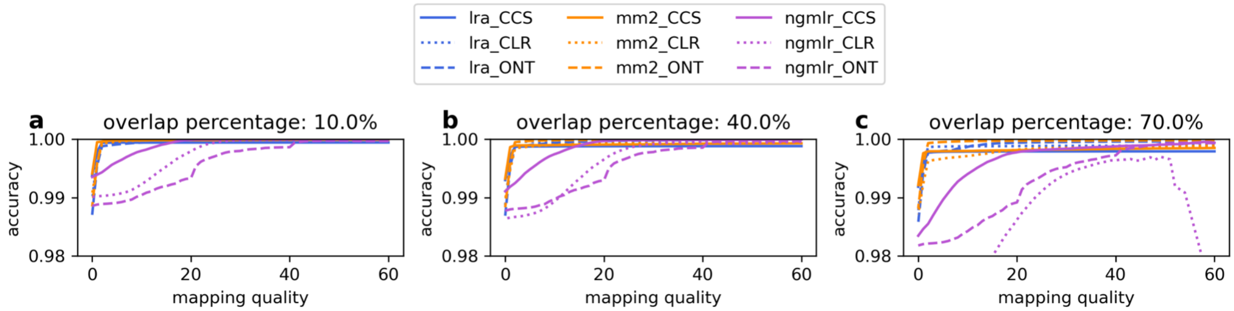

COMMENT 18.
6. The authors do not provide any alignment quality comparison for the real data with lra. The significance of CG-SDP may not be captured with the simulated data since the gaps simulated may not reflect the biological variance. However, an analysis of the alignment algorithm for the real reads data can be useful.

RESPONSE 18.
While the analysis of our method for Figure 1 uses simulated data in order to have ground truth for placement of alignments, the analysis presented across all tables and Figure 2 are on real data summarized in response 15. We evaluate the placement of gaps using the Genome in a Bottle gold standard callset.

COMMENT 19.
7. The results focuses on the number of SV calls detected using Ira but an analysis about the accuracy of the SV breakpoints using Ira can also be useful since Sniffles is capable to make precise breakpoint calls for SVs.

RESPONSE 19.
We have added breakpoint accuracy analysis on simulated reads against SURVIVOR simulated variants, and on real reads with the Genome in a Bottle truth set. We have added the following analysis of Truvari true positive SVs' breakpoints to the manuscript:

> The average shifting distances of all combinations of SV calling algorithms for Ira is 53 across all data type and SV calling algorithms, compared to an average shifting distance of 46 bases across all aligner/data/SV calling methods. The percentages of SVs with perfect breakpoints called from Ira alignments ranged between 31-64% across all data and SV calling algorithms (Supplementary Table S9), which indicates in addition to alignment, the accuracy of breakpoints also depends on SV caller.

COMMENT 20.
8. Ira shows an improvement for the SV calls mainly done for the insertion and deletion type variants. However, Sniffles is capable of capturing other complex nested variants. An analysis of such nested variants using Ira can be useful. Also, an analysis of the SV callsets and the breakpoint accuracy on the simulated variant reads data can be useful (for both large indel variants as well as other complex and nested variants).

RESPONSE 20.
We have added SURVIVOR simulations of insertion/deletion, inversion, deletion-inversion-deletions, and inverted duplications. Extensive results for the simulations are added to the supplementary material.

To discuss simple SV, we have added the following text to the manuscript:

> Both pbsv and cuteSV calling results show similar f1 score (>= 0.974, 0.979, 0.974) for Ira, minimap2 and ngmlr alignments on insertions and deletions over all data types. All three aligners achieved comparable f1 scores (>= 0.97) on inversions from cuteSV calling results over all data types (Supplementary Table S7).

To discuss nested SV, we have added the following text to the manuscript:

> Complicated nested SVs like INVDEL presented a greater challenge, particularly for lower accuracy reads. Using simulated HiFi reads, 98 of the INVDEL and 94 of the INVDUP were correctly called by Ira, with all alignment methods detecting at least 93 variants in each data set. The majority of the INVDEL variants detected by Ira alignments

> using CLR (79) data missed one deletion, while variant calls from minimap2 and ngmlr correctly detected 94 and 74 INVDEL variants, respectively. A different pattern existed for ONT data, where 1-2 correct calls were detected from minimap2/ngmlr, and 37 by Ira. The INVDUP calls were successfully detected from Ira and ngmlr alignments, and represented as insertions by minimap2. The Ira alignments resulted in 99 and 100 calls from the CLR and ONT simulated reads, and ngmlr 98 calls from both datasets (Supplementary Table S8).

We have added breakpoint analysis of the simulated data to the supplementary material.

COMMENT 21.

9. The results shows that F1 score is better for SV discovery using read data alignment than contig alignments. However, the importance of the contig alignments in SV discovery is not clear? It will be useful if the authors can emphasize on it. Are the contig alignments capable of detecting SVs that are difficult to detect using the alignments of the reads data? If so, a comparison of the unique SV calls made using both methods should be added to the results.

RESPONSE 21.
We note that we have switched to the hifiasm assembly recently released on HG002. The contiguity is vastly superior to other assemblies we have used, and has an increased F1 score (.955 versus .938), however this is less than what we currently have with the Ira HiFi/pbsv calls (.97).

We have added a sentence addressing the difference between F1 scores of the assembly and HiFi callsets. The majority of differences are likely due to shifted breakpoints or the merging step used to generate the diploid vcf, and finally areas where a haplotype was assembled into both copies.

> There are 233 calls from the Ira assembly callset missing from the true-positive annotations of the Ira HiFi/pbsv callset. Upon inspection, the majority of these missed calls are due to a combination of the process used to merge haplotype calls into a diploid callset, shifted breakpoints relative to the GIAB annotation, and haplotype dropout/swapping of de novo assembly sequences.

We have added an additional paragraph describing the extent of the genome that is covered relative to the read-based callsets. Strikingly, effectively all of the genome where calls may be made from reads are covered by the de novo assembly. We also catalog up to 60 long (>20kb) variants per haplotype that are observed from the assemblies that are missing from the read callsets.

We have added the following paragraph:

Diversity studies are increasingly using SMS de novo assemblies to measure variation (audano 2019 characterizing,ebert 2021 haplotype) rather than from sequencing reads. An integrated analysis of the regions of the genome spanned by assembly alignments and the raw-read base calls from multiple alignment and SV discovery methods can help reveal the extent that variation is discovered and potentially missing from a high-quality haplotype-resolved de novo assembly. Across all read technologies, alignment algorithms, and variant discovery methods, 167 unique calls representing 0.6% of an average read-based SV callset were in regions not mapped by either haplotype of the assembly. Of these, 113 are in segmental duplications (SD). Because SDs make up roughly 5% of the human genome, the majority of SD regions that are mapped by SMS reads in this study are assembled in the hifiasm genome. The lra alignments detect 53 and 60 large SV (>20kb) totaling over 3Mb from the haplotype 1 and haplotype 2 assemblies that are not found in the read SV callsets (Supplementary Table S2, S4). Because the length of SV negatively correlates with allele frequency, and is associated with enrichment in genome-wide association studies significant loci (sudmant 2015 integrated), these larger variants represent a class of variation that is biologically important for discovery.

COMMENT 22.

10. The significant improvement in SV call count for ONT data are partially attributed to increasing recall for large insertions. How efficient is the algorithm in making the distinction between large insertions and large deletions? Perhaps an analysis using the simulated variants can be useful.

RESPONSE 22.

The CG-SDP algorithm considers gaps as deviations from a diagonal, and has equal penalty for insertion versus deletion gaps. Insertion calls are largely limited by the read length. On CCS and CLR data, 100% of both insertion and deletion data, including calls up to 10kb.
